# Lack of Antibodies to SARS-CoV-2 among Blood Donors during COVID-19 Lockdown: A Study from Saudi Arabia

**DOI:** 10.3390/healthcare9010051

**Published:** 2021-01-05

**Authors:** Thamir A. Alandijany, Sherif A. El-Kafrawy, Abrar A. Al-Ghamdi, Fadi S. Qashqari, Arwa A. Faizo, Ahmed M. Tolah, Ahmed M. Hassan, Sayed S. Sohrab, Salwa I. Hindawi, Maha A. Badawi, Esam I. Azhar

**Affiliations:** 1 Special Infectious Agents Unit, King Fahd Medical Research Center, King Abdulaziz University, P.O. Box 128442, Jeddah 21362, Saudi Arabia; saelkfrawy@kau.edu.sa (S.A.E.-K.); aayalghamdi@kau.edu.sa (A.A.A.-G.); aafaizo@kau.edu.sa (A.A.F.); atoulah@kau.edu.sa (A.M.T.); msahmed@kau.edu.sa (A.M.H.); ssohrab@kau.edu.sa (S.S.S.); 2 Department of Medical Laboratory Technology, Faculty of Applied Medical Sciences, King Abdulaziz University, P.O. Box 80324, Jeddah 21589, Saudi Arabia; 3 Department of Microbiology, Faculty of Medicine, Umm Al-Qura University, P.O. Box 715, Makkah 21955, Saudi Arabia; fsqashqari@uqu.edu.sa; 4 Department of Hematology, Faculty of Medicine, King Abdulaziz University, P.O. Box 3646, Jeddah 22252, Saudi Arabia; shendawi@kau.edu.sa (S.I.H.); mbadawi2@kau.edu.sa (M.A.B.); 5 Blood Transfusion Services, King Abdulaziz University Hospital, P.O. Box 3270, Jeddah 22252, Saudi Arabia

**Keywords:** COVID-19, SARS-CoV-2, Saudi Arabia, blood donors, seroprevalence, ELISA, antibodies, lockdown

## Abstract

In response to the coronavirus disease 2019 (COVID-19), Saudi Arabia have imposed timely restrictions to minimize the infection spread, lower the risk for vulnerable groups, and reduce the pressure on healthcare services. The effectiveness of these measures has not been assessed comprehensively and, thereby, remains uncertain. Besides monitoring the number of COVID-19 cases diagnosed by molecular assays, the seroprevalence can serve as an indicator for the incidence rate among the general population. This study aimed to evaluate seroprevalence status of all healthy blood donors who attended one of the main largest hospital located in the western region of Saudi Arabia from 1 January to 31 May 2020. The study period covered two months prior to reporting the first COVID-19 case in the country on 2 March 2020. Importantly, it covered the period when “lock-down type” measures have been enforced. Samples were subjected to in-house enzyme-linked immunosorbent assay (ELISA), chemiluminescence immunoassay (CLIA), and microneutralization (MN). The sero statuses of all samples were confirmed negative, demonstrating the lack of antibodies to severe acute respiratory syndrome coronavirus 2 (SARS-CoV-2) among blood donors during COVID-19 lockdown period. This study supports the hypothesis that COVID-19 restrictions have potential for limiting the extent of the infection.

## 1. Introduction

The coronavirus disease 2019 (COVID-19) continues to pose a global threat to the human population. The first cases of COVID-19 were reported from Wuhan, China in late December 2019 [1]. Since then, the infection has spread worldwide leading to more than 60 million cases and 1.5 million deaths as of 30 November 2020 [2].

Officials in the affected countries have adopted varying strategies to deal with this crisis [3,4,5,6,7]. In Saudi Arabia, policymakers have been proactive in their response to COVID-19 and imposed control measures (e.g., evacuation of Saudis from China, suspension of flights from and to COVID-19 hit countries including China, and suspension of Umrah and tourism) during February 2020 [3,8,9]. These measures were imposed even before reporting the country’s first case on 2 March 2020 by the Ministry of Health (MOH) [10]. This was followed by a series of further unprecedented decisions including suspension of local and international flights, closure of schools and universities, curfews, lockdown of some cities and the holy mosques, and provision of free healthcare to COVID-19 patients [3,8,9]. The MOH announced a three-stage plan to return to “normal” life starting from 28 May to 21 June 2020 [11]. As of now (the end of December 2020), some COVID-19 measures are still enforced such as maintaining social distancing, wearing of face masks in public place, and activation of E-learning. These continuing sustained efforts are thought to have been crucial to initially limiting the massive spread of infection. However, the effectiveness of these measures remains unclear because this issue has not been investigated.

Molecular and serological tests are valuable tool for estimating virus circulation among populations [12,13]. Serology is particularly important in the case of COVID-19, as many infected individuals mount antibody response but remain asymptomatic during the course of the infection [14,15,16]. Furthermore, the seroprevalence rate provides an indication of herd immunity status.

Several reports, from different countries, have investigated the seroprevalence status of their population [17,18,19,20,21,22]. Apart from a single study conducted on healthcare workers [23], there is a lack of report about the seroprevalence status of the Saudi population. Utilizing three serological assays, we investigated the seroprevalence of anti-SARS-CoV-2 antibodies among healthy blood donors who attended a >1000-bed hospital located in Jeddah, the western region of Saudi Arabia from 1 January 2020 to 31 May 2020. Initially, all samples were screened for anti-SARS-CoV-2 antibody by a recently optimized in-house enzyme-linked immunoassay (ELISA) [24]. This assay offers 100% sensitivity and 98.4% specificity [24]. Hence, all samples that tested positive were subjected to microneutralization (MN) assay and commercial chemiluminescence immunoassay (CLIA) for confirmation. This study aimed to (1) identify any cases of COVID-19 prior to the first case officially reported in the country (case zero), and (2) assess the extent of infection spread during the implementation of COVID-19 restrictions and lockdown.

## 2. Materials and Methods

### 2.1. Ethic Statement

The Research Ethics Committee (REC) of the unit of biomedical ethics located at the Faculty of Medicine, King Abdulaziz University, Jeddah, Saudi Arabia has approved this study (reference no. 487-20).

### 2.2. Study Population

Participants of this study were all healthy blood donors (n = 956) who attended the King Abdulaziz University Hospital (KAUH), Jeddah, Saudi Arabia from 1 January 2020 to 31 May 2020 and were eligible for donation. All blood donors were aware that their samples might be used for research purposes and written consent forms were obtained.

### 2.3. Immunoassays

#### 2.3.1. In-House Enzyme-Linked Immunoassay (ELISA)

Sera of blood donors were screened for anti-SARS-CoV-2 IgG antibodies using our recently developed in-house ELISA [24]. Microtiter plates (Immulon^®^ 2 HB, Bloomington, Minnesota, USA) were coated with 100 ng per well of SARS-CoV-2 spike recombinant protein (Sino Biological, Beijing, China). Following three washes with PBST (PBS containing 0.1% Tween 20), blocking was performed in PBST containing 5% skimmed milk (blocking buffer). Three washes with PBST were performed prior to addition of samples. Sera were diluted at 1:100 in blocking buffer, added, and incubated for an hour at 37 °C. The plates were washed three times with PBST. Secondary antibody (goat KPL peroxidase-labelled antibodies to human IgG, Seracare, Milford, MA, USA) at a dilution of 1:64,000 in PBST was added and incubated for an hour at 37°. Three more washes were conducted. Then, 3,3′,5,5′-tetramethylbenzidine (TMB) (Seracare, USA) was added for 5 min before stopping the reaction using hydrochloric acid (HCL). The optical density was measured using an Elx 800 bioelisa Reader (Biokit, Barcelona, Spain) at 450 nm (OD450) with OD450 values of ≥0.27 considered to be positive. Under these experimental conditions, the assay provides 100% sensitivity (no false negative) and 98.4% specificity (minimal false positive) [24]. Samples from COVID-19 recovered patients were included as positive controls. All samples were run in triplicate. Those that tested positive were subjected to CLIA and MN assay (the gold standard), as described below.

#### 2.3.2. Chemiluminescence Immunoassay (CLIA)

Samples that tested positive by ELISA were subjected to commercially available CLIA (VITROS Immunodiagnostic Products Anti-SARS-CoV-2 IgG Reagent Pack, Reference 619 9919), according to manufacturer instructions.

#### 2.3.3. Microneutralization (MN) Assay

The MN assay, the gold standard for antibody detection, was conducted, as previously described [24]. African green monkey kidney cells Vero E6 (ATCC^®^ CRL-1586™) were utilized to propagate and titrate the local SARS-CoV-2 clinical isolate (SARS-CoV-2/human/SAU/85791C/2020) (Genbank accession number MT630432.1). Inactivation of serum samples was conducted at 56 °C for 30 min. Serially diluted samples plus 100 TCID50 of SARS-CoV-2 were added on Vero E6 cells and incubated at 37 °C in 5% CO_2_ for 3 days. Positive sample had MN titer of ≥1:20.

### 2.4. Data Curation

GraphPad Prism software version 9 (GraphPad Software, La Jolla, CA, USA) was used for data curation.

## 3. Results

### 3.1. COVID-19 Status in SAUDI Arabia during the Study Period (1 January to 31 May 2020)

**Figure 1:**
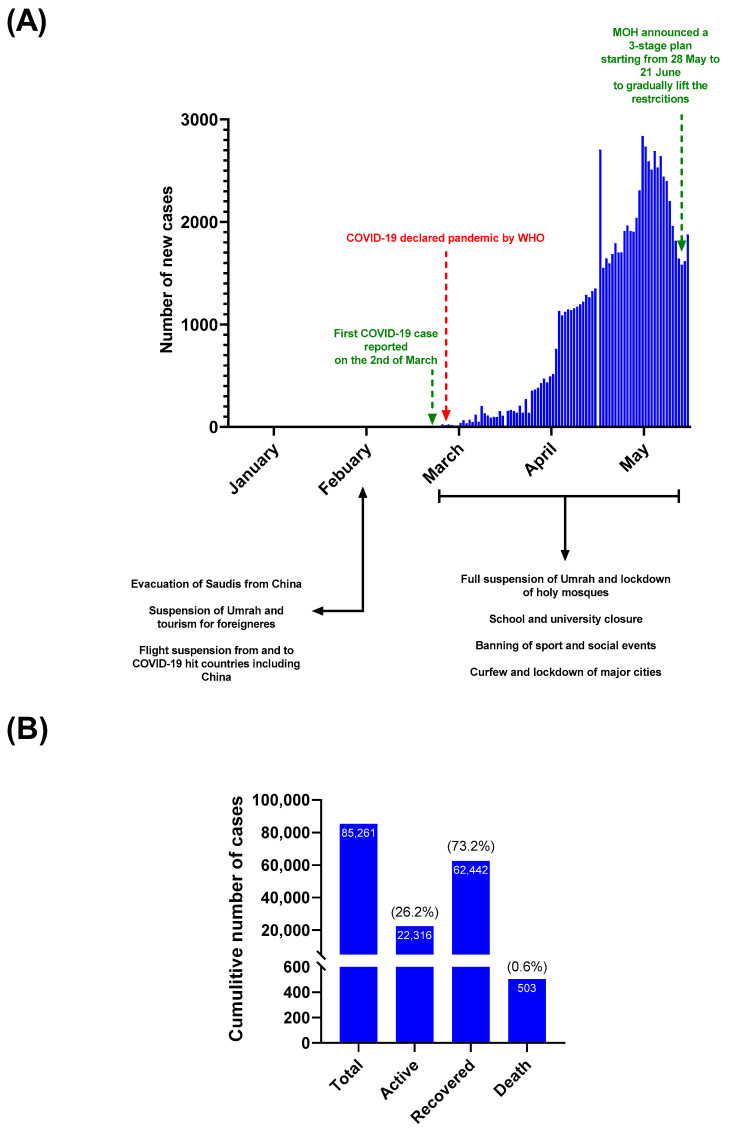
COVID-19 status in Saudi Arabia by the end of May 2020. (**A**) Demonstrates the number of new cases reported by the Ministry of Health (MOH) from 1 January to 31 May 2020. Some key events (e.g., country’s first case, initiation of control measure, and lifting of restriction) during this period are indicated; (**B**) Shows the cumulative numbers of cases, active cases, recoveries, and deaths by the end of May 2020. Percentages of active, recovered, and death relative to total number of cases are indicated.

Despite the proactive response from Saudi Arabia to COVID-19, the MOH officially reported the first case on 2 March 2020. That is over three months from the first case discovered in Wuhan, China, and only a few days before declaring COVID-19 as a pandemic by the World Health Organization (WHO) [25]. Since then, the number of cases has continued to escalate despite further control measures imposed by the government. By the end of May 2020, the cumulative numbers of cases and deaths were 85,261 and 503, respectively [2,26]. These represent ~2507 cases and ~14.8 deaths per million population. The recovery rate has been promising, with 62,442 reported recoveries (73.2%) [2,26] (Figure 1). It is important to note that all of these numbers were based on PCR-based diagnosis with most cases reported from the western and central region of Saudi Arabia [26].

### 3.2. The Seroprevalence of SARS-CoV-2 among Blood Donors

#### 3.2.1. Screening of Sera by In-House ELISA

**Figure 2:**
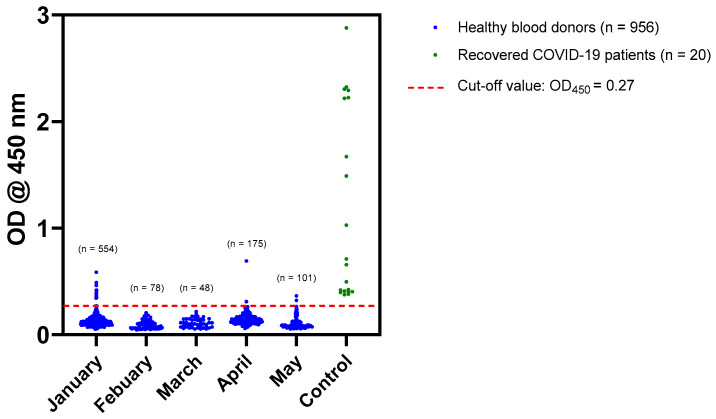
In-house ELISA screening of the seroprevalence status among blood donors. The optical density value at 450 nm (OD450) obtained from each sample is shown (blue). Samples from COVID-19 recovered patients were utilized as controls (green). The cut-off value of the assay is 0.27 (red dashed line). Fourteen samples tested ≥0.27 and were considered to be positive. All samples were run in triplicate. Means of OD450 values for each sample are shown.

All blood donors who visited KAUH, Jeddah, Saudi Arabia from 1 January 2020 to 31 May 2020 were included in this study. The total number of samples was 956. The number of donors varied considerably from month to month, i.e., January (n = 554), February (n = 78), March (n = 48), April (n = 175), and May (n = 101). Overall, these numbers are substantially lower than usually due to the control measures implemented in response to COVID-19 pandemic. Utilizing in-house ELISA, all sera were screened for anti-SARS-CoV-2 IgG antibodies. This assay offers 100% sensitivity and 98.4% specificity when previously evaluated against MN assay **^24^**. Out of the 956 samples, 942 samples tested negative (OD_450_ < 0.27). The OD_450_ of the remaining 14 samples were ≥0.27 and considered to be positive (Figure 2).

#### 3.2.2. Confirmation of Sero Status by CLIA and MN Assay

**Figure 3:**
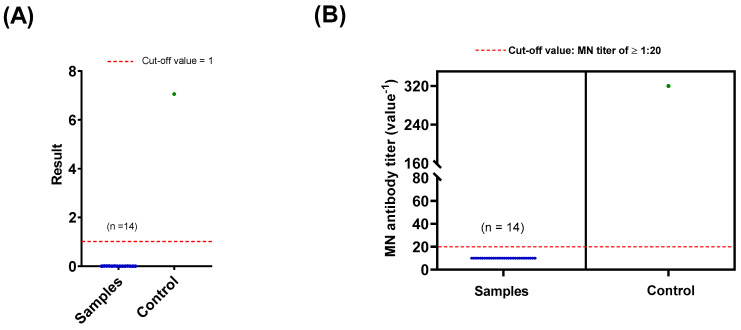
The seroprevalence status of blood donors by chemiluminescence immunoassay (CLIA) and microneutralization (MN) assay. Fourteen samples tested positive by in-house ELISA were further subjected to CLIA and MN assay ((**A**) and (**B**), respectively). Red dashed lines represent the cut-off value of each assay. All samples were tested negative by both assays (blue). A sample from COVID-19 recovered patients was utilized as control (green).

Given that our in-house ELISA is a screening tool, those 14 samples tested positive were subjected to two more serological assays (CLIA and MN assay) to confirm their statuses. MN assay is the gold standard for determining the presence of SARS-CoV-2 antibody. All samples were tested “non-reactive, <1” by CLIA (Figure 3A). Their MN titers were also below 1:20 which represents the cut-off value of MN assay (Figure 3B). In all experiments, samples from COVID-19 recovered patients were included as positive controls. Collectively, these results demonstrate that all blood donors during the study period (1 January to 31 May 2020) were negative for anti-SARS-CoV-2 IgG antibodies.

## 4. Discussion

Many countries, including Saudi Arabia, implemented several control measures and restrictions in response to COVID-19 pandemic (Figure 1) [3,4,5,6,8,9]. The effectiveness of this strategy to limit the spread of the infection remains unclear and needs further investigation [6,7]. Here, we reveal the absence of sero-positivity among healthy blood donors during the “lock-down type” measures in Saudi Arabia, which is indicative of potentially efficient control of virus circulation.

Initially, all samples were screened for anti-SARS-CoV-2 antibody by a recently optimized ELISA [24]. This assay offers 100% sensitivity, and high overall accuracy and reproducibility, validating it as a valuable screening tool [24]. The vast majority of samples (n = 942 out of 956) tested negative (Figure 2). Although a few samples (n = 14) tested positive by in-house ELISA, the status of these samples were confirmed to be negative by CLIA and MN assay (Figure 2 and Figure 3). It is important to note that MN assay is the gold standard for evaluating the presence of antibody and, thereby, used as a confirmatory assay [27]. The false positive results obtained from the in-house ELISA could be due to unspecific binding to the viral antigen or the presence of interfering substances in the samples. In fact, we have previously reported that our assay provides 98.4% specificity which corresponds to the data reported here [24].

To our knowledge, this is the first investigation addressing the seroprevalence status of SARS-CoV-2 among general population. A recent national study conducted on healthcare workers demonstrated an overall seroprevalence rate of 2.37% [23]. The rate considerably varied among regions/cities with the highest rate observed among healthcare worker from the western region (6.31% of 2,678). The study period was between 20 and 30 May 2020 [23]. In this study, we have not identified any positive cases among healthy blood donors from 1 January to 31 May 2020. This can be explained by the fact that healthcare workers are at increased risk of acquiring the infection due to their potential close contact with COVID-19 patients.

In summary, we have not identified any sero-positive cases among blood donors screened in this study. Our data suggest that COVID-19 restrictions and control measures played an effective role in limiting the initial spread of the infection. It is also indicative of the low herd immunity status during the study period. This observation is interesting as compared with reports from other countries (e.g., Iran, Brazil, and USA) where high seroprevalence rates have been reported [20,21,22]. Variation between studies can be due to differences related to study population, sample size, study period, antibody detection methods, and countries’ strategies for dealing with COVID-19 pandemic. It is important to note that the data presented here were obtained from a single hospital. Further assessment of a large nationwide sample is required to fully comprehend the national seroprevalence status, whether during or after COVID-19 restriction period. Indeed, it is likely that the seroprevalence rate among general population in the country would be higher after restrictions were relaxed. In addition, here, we only investigated the prevalence of IgG antibody to SARS-CoV-2. Several reports have demonstrated the rapid seroconversion of infected individuals with high detection probability of IgG antibodies [28,29]. Yet, without molecular technique and screening of IgM, it remains possible to miss a few recently infected individuals.

## Data Availability

The data that support the findings of this study are available from the corresponding author, upon reasonable request.
